# Death Education for Palliative Psychology: The Impact of a Death Education Course for Italian University Students

**DOI:** 10.3390/bs13020182

**Published:** 2023-02-16

**Authors:** Lucia Ronconi, Gianmarco Biancalani, Georgiana Alexandra Medesi, Hod Orkibi, Ines Testoni

**Affiliations:** 1 IT and Statistical Services, Multifunctional Pole of Psychology, University of Padova, Via Venezia, 8, 35131 Padova, Italy; 2 Department of Philosophy, Sociology, Education and Applied Psychology (FISPPA), University of Padova, 35131 Padova, Italy; 3 Drama & Health Science Lab, and the Emili Sagol Creative Arts Therapies Research Center, Faculty of Social Welfare and Health Sciences, University of Haifa, Haifa 3498838, Israel

**Keywords:** death education, palliative psychology, university students, psychodrama, photovoice

## Abstract

The present study investigated the effects of a hybrid online course on a group of Italian Master’s degree students involved in a European Erasmus+ project. The course was composed of nine modules about death education, palliative psychology and the use of creative arts therapies—such as psychodrama, intermodal psychodrama and photovoice—in the end-of-life-field. The project involved 64 students in the experimental group (who attended the course) and 56 students as the control group. Both groups completed an online questionnaire before and after the delivery of the course and 10 students from the experimental group participated in a focus group at the end of the course. The quantitative analysis revealed that the experimental group students showed lesser levels of perception of death as annihilation, fear of the death and death avoidance, while they increased their levels of death acceptance, creative self-efficacy and attitude toward the care of the dying. Qualitative analysis identified three main themes: the positive impact of the course on death education and end-of-life care; the role of art therapies on death and end-of-life care; and the unhelpful facets of the course. Overall, this intervention changed the perception and the feelings of the students regarding the themes of death and palliative psychology and increased their creative self-efficacy and their interest in working in an end-of-life field.

## 1. Introduction

The term death education denotes a set of activities that aim to promote reflection, facilitate understanding and the processing of dysfunctional emotions on existential issues concerning death, dying and bereavement [1,2]. The objectives of these activities are to provide general information about death and dying through simple and appropriate language, to create a space for reflection on the meaning of life and death, to strengthen critical thinking, and to stimulate discussion on feelings of anxiety, suffering and fear provoked by the thought of death and dying [2,3]. Death education interventions are delivered at three different levels: 1. Primary: this encompasses educational interventions in which the topic of death is addressed with participants who are not personally or closely experiencing death, and sharing the related emotions; 2. Secondary: when accompanying a person to death, providing support to the bereaved and their loved ones; 3. when the bereavement of a missing person is processed [4,5].

The literature shows that death education interventions are relevant because they reduce the attitudes of fear or death avoidance [6,7,8,9,10,11], help participants to develop positive attitudes toward death and dying and allow participants to view death as a natural transition at the end of life [9,12]. Death education interventions have also been proven to be effective in fostering the recognition and expression of feelings related to the topic of death [3,4,9] and in strengthening expressive and creative skills, when associated with art therapies [12,13]. Discussing the topic of death in a welcoming and non-judgmental environment provides individuals with more effective language to adequately express the emotions related to this topic [3,9] and increases empathic understanding [14,15]. Discussing one’s mortality can motivate people to improve their physical and mental health, prioritize growth-oriented goals, and increase life satisfaction [16,17].

Among the methods used in death education interventions, the literature indicates that creative arts therapies have proven to be effective in promoting participants’ psychological well-being [9,18,19]. In particular, the literature shows that the use of creative arts therapies in death education interventions brings beneficial effects regarding the recognition of the end expression of emotions related to the themes of death and dying, to better manage fear and death anxiety and the grieving process [9,18,20]. The term arts therapies encompasses multiple methods of psychological support/psychotherapy: art therapy, dance/movement therapy, drama therapy, psychodrama, music therapy and bibliotherapy [21]. By fostering creative expression, creative arts therapies succeed in enhancing the well-being of participants by providing adequate psychological support [22,23].

Previous studies have shown that the use of psychodrama in death education has proven to be effective in the processing of dysfunctional emotions related to talking about the topic [8,12] anticipatory grief [18], and bereavement processing [24,25]. In addition, psychodrama has been shown to be useful in improving participants’ communicative and descriptive skills regarding feelings related to death [9,18], bringing participants’ attention to their own inner dimensions [9]. The term psychodrama indicates a method devised by J. L. Moreno used in psychotherapy and psychological support that uses dramatization to explore the emotions and personal or collective experiences of the participants’ inner world [26,27]. Through this method, the participant is engaged in listening to the different parts of his/her internal world and relationship, initiating an internal dialogue that guides him/her to discover possible solutions to his/her intrapsychic and/or relational conflicts with the external world [28], re-experiencing past experiences and reaching a higher level of consciousness and self-knowledge [29]. When psychodrama is combined with other creative arts therapies, it is called intermodal psychodrama [30]. One example is the CBN psychodrama model that integrates visual art making with cognitive behavioral therapy and narrative therapy [31]. A second example is the Kinetic Family in Action assessment method that integrates drawing, writing and role playing [32].

Another artistic method used effectively in death education interventions is photovoice [5,9]. Photovoice uses photography as an expressive and communicative channel, with the aim of increasing knowledge and understanding of a given social phenomenon and to integrate the knowledge gained with interventions of a political, or social nature or, in general, for the benefit of community members [33,34]. In this method, participants are asked to express their perspectives, views, and feelings toward a research topic through photography. First, the participants take a picture on a certain theme or topic, and associate a short caption to their photo. Subsequently, they discuss the taken photo and the given caption as a group and can choose the most representative photo from those in the group, possibly changing the caption and elaborating on further dialogues on the research topics [33,34].

The literature confirms that the use of photovoice in death education interventions increases the level of wellbeing and self-awareness in participants [12,35], contributes to a decrease in the fear of death and a more positive attitude towards death, and fosters a greater understanding and expression of feelings related to bereavement and managing death anxiety [5,9,35].

The aim of the present study was to evaluate the impact of a death education and palliative psychology course, associated with art therapy activities, on Psychology master’s degree students from the University of Padova (Italy). In particular, the impact of the course on the perception and vision of death was assessed, and it was investigated whether psychodrama and photovoice could represent effective clinical tools within death education and palliative psychology courses and the change in professional interest towards the field of palliative care was evaluated.

## 2. Materials and Methods

### 2.1. Study Design

The study followed an explanatory sequential mixed methods design, that consisted of a first quantitative research phase in which the quantitative data were collected and analyzed, followed by a qualitative research phase to explain the qualitative results in more detail [36]. The quantitative phase consisted in the administration of an online questionnaire to the experimental group before and after the project intervention and at the same time to the control group. The qualitative phase consisted in the participation of 10 students in the experimental group in a focus group, which took place after the participation in the project intervention.

### 2.2. DE4PP Project Intervention

Death Education for Palliative Psychology (DE4PP) is an Erasmus+ project, Co-funded by the Erasmus+ Program of the European Union, that involved the University of Padova in Italy and another four Universities in Austria, Israel, Poland and Romania. Italian Onlus Assistenza Nazionale Tumori—ANT (National Tumor Assistance) was also involved in the project as a partner. ANT provides medical assistance to cancer patients who are often terminally ill. The DE4PP project had the aim of educating university students on the topics of death education and palliative psychology, teaching them the theoretical bases of these topics and using the arts therapies techniques and psychodrama to psychologically support people at the end of their life and their families. To do so, a group of Italian students took part in an online pilot course on a specially created Moodle platform. To access the online course, the students’ email addresses were collected and they were enrolled in the Moodle platform where they could follow the lessons. The course consisted of nine modules, each containing three activities: a recorded video lesson lasting about 20 min, the PDF document of the video lesson slides and a three-question knowledge questionnaire to evaluate their understanding of the contents of the video lesson. To move onto a subsequent module, each student had to complete the previous ordered activities first. The first five modules focused on the explanation of death education and palliative psychology, in particular, the theoretical basis of death education and palliative care, the communication in palliative care, the advanced care planning, and how to provide psychological support to patients. The last four modules focused on the use of arts therapies in the field of death education and palliative psychology. The considered arts therapies were: psychodrama, intermodal psychodrama and photovoice. In detail, these were phototherapy in death education, psychodrama for the management of death, intermodal and CBN psychodrama with bereaved adults, and psychodrama for psychological support for caregivers working with end-of-life patients. At the end of each of these last four modules, and before starting the next one, the students took part in a three-hour applied training workshop on the use of the arts therapies that was illustrated in the online lesson in an end-of-life setting. The workshops were conducted in person by one of the authors inside a university classroom. In particular, the first workshop dealt with the explanation of a photovoice activity that the students should have done in groups of five before the end of the course. Each student of the group was asked to take a picture and give it a caption inherent to the topics of death education and/or palliative psychology, then inside the group each caption was read and eventually improved. Finally, each group selected the best picture and created a written document containing the selected picture with its caption, the description of what it represented, the theoretical background it referred to and also, at the bottom, all the other four pictures. The second workshop was about the use of the psychodramatic technique of the social atom in dealing with the topic of death; the third workshop showed the use of intermodal and CBN psychodrama to provide emotional support to grieving adults; and the fourth workshop was a psychodrama session to provide psychological support to people who work in contexts in which they have contact with death.

At the end of the course, each group of students had to submit their photovoice activity and the group that had done the best work was selected to go to the ANT headquarters in Bologna (Italy) to attend a three-day educational experience. This consisted of: explaining the work of the palliative care team and the palliative psychologist; a role-playing activity regarding the doctor-patient approach at the end of life; and a day when students accompanied an ANT doctor in his job of providing home care assistance to the terminally ill patients.

### 2.3. Participants

The participants were master’s students of psychology at the University of Padova (Italy). There were a total of 139 at the first administration of the questionnaire, 71 from the experimental group students and 68 from the control group students. A total of 120 students completed both the pre- and post- questionnaires; 64 students from the experimental group and 56 from the control group. Additional characteristics of the participants are shown in Table 1.

Among the experimental group students, ten students (nine female and one male) were involved to participate in the focus group held after the end of the course. The participation in the focus group was on a voluntary basis, and since 88% of the participants in the experimental group were female, a high number of female participants was also in the qualitative sample. Five of these participants were only students, while the other five were student workers, meaning people who are currently enrolled in an educational institution while working part-time or full-time. Four of them were below 25 years old, four of them had an age between 25 and 30 years old and two were older than 30 years old.

### 2.4. Recruitment

Experimental group students were recruited by convenience sampling. They were interested students recruited in the university courses of the professor who was part of the DE4PP project, and they decided to freely participate in the online course offered by the DE4PP project; non-participation carried no penalty.

Students in the control group were selected through snowball sampling, asking students in the experimental group for the names of psychology students from the same University who could be contacted by the researchers to be part of the control group.

Furthermore, at the end of the pilot course, 10 students were randomly selected among those who were part of the experimental group to take part in a two-hour focus group.

### 2.5. Data Collection

#### 2.5.1. Quantitative Data Collection

Quantitative data were collected through an online questionnaire developed on Qualtrics lasting about 20 min. The experimental group found the two links of the questionnaire within the Moodle platform (one link at the beginning of the course and one at the end). The control group received the questionnaire link via email by the researchers.

The experimental group completed the questionnaire at two separate times: at the beginning and at the end of the online course. At the same two times as the experimental group, the control group students also completed the questionnaire.

#### 2.5.2. Qualitative Data Collection

Qualitative data was collected through a focus group lasting two hours. The focus group was conducted online, on the Zoom platform, by a psychologist expert in the topics covered and who was aware of the project structure. The selected students received the link to access the online focus group via email from the conductor. The entire session was recorded and then transcribed verbatim, and the texts analyzed.

### 2.6. Instruments

#### 2.6.1. Quantitative Measurement

Testoni Death Representation Scale (TDRS) [37]: is a six-item five-point Likert scale (1 = strongly disagree, 5 = strongly agree) that investigates the representations of death as a passage or annihilation. The scale was validated in the Italian language, and an example of the item is as follows: “Death is only a passage. After I die, I will continue to exist and will remember this life’s experiences.” The internal consistency reliability of the scale is good both in the pre-test (Cronbach’s alpha = 0.88) and in the post-test (Cronbach’s alpha = 0.87).

Death Attitude Profile- Revised (DAPR) [38] is a thirty-two--item five-point Likert scale (1 = strongly disagree, 5 = strongly agree) that investigates attitudes toward death. It is composed of five factors: Fear of Death, Death Avoidance, Neutral Acceptance, Approach Acceptance, and Escape Acceptance. The present study used the Italian validation of the scale [39]; and an example of the item is as follows: “Death is no doubt a grim experience.” The internal consistency reliability of the Fear of Death subscale is good both in the pre-test (Cronbach’s alpha = 0.88) and in the post-test (Cronbach’s alpha = 0.89). The internal consistency reliability of the Death Avoidance subscale is excellent both in the pre-test (Cronbach’s alpha = 0.92) and in the post-test (Cronbach’s alpha = 0.93). The internal consistency reliability of the Neutral Acceptance subscale is poor in the pre-test (Cronbach’s alpha = 0.59) and questionable in the post-test (Cronbach’s alpha = 0.69). The internal consistency reliability of the Approach Acceptance subscale is excellent both in the pre-test (Cronbach’s alpha = 0.93) and in the post-test (Cronbach’s alpha = 0.94). The internal consistency reliability of the Escape Acceptance subscale is good in the pre-test (Cronbach’s alpha = 0.81) and excellent in the post-test (Cronbach’s alpha = 0.90).

Career Commitment Scale (CCS) [40]: is an eight-item five-point Likert scale (1 = strongly disagree, 5 = strongly agree) that measures and predicts professional commitment and vocation; it was specifically modified for end-of-life care. The items were modified by adding a reference to the course, for example: “I definitely want a career for myself in end-of-life care.”

The Italian translation indicated a good internal consistency reliability both in the pre-test (Cronbach’s alpha = 0.85) and in the post-test (Cronbach’s alpha = 0.89).

Creative Self-Efficacy Scale (CSE) [41] is a five-item five-point Likert scale (1 = strongly disagree, 5 = strongly agree) that measures an individual’s self-belief in their ability to be creative when required by a given situation. The items were modified by adding a reference to the course, for example: “I am good at coming up with new ideas for arts-based end-of-life care.” The Italian translation indicated an excellent internal consistency reliability both in the pre-test (Cronbach’s alpha = 0.97) and in the post-test (Cronbach’s alpha = 0.97).

Frommelt Attitude Toward Care of the Dying Scale—Form B. (FATCOD) [42] is a thirty-item five-point Likert scale (1 = strongly disagree, 5 = strongly agree) that measures the attitudes of medical and psychological personnel regarding the care of dying patients. The present study used the Italian validation of the scale [43]; an example of the item is as follows: “Giving care to the dying person is a worthwhile experience.” The internal consistency reliability of the scale is acceptable in the pre-test (Cronbach’s alpha = 0.79) and good in the post-test (Cronbach’s alpha = 0.84).

Compassion Scale (CS) [44] is a sixteen-item five-point Likert scale (1 = strongly disagree, 5 = strongly agree) that measures the psychological construct of compassion, understood as kindness, a sense of humanity, mindfulness, and attention to the suffering of others. In the present study only two subscales were used: Kindness (four items) and Indifference (four items); an example of the item is as follows: “I am unconcerned with other people’s problems.” The Italian translation indicated an acceptable internal consistency reliability for the Kindness subscale both in the pre-test (Cronbach’s alpha = 0.79) and in the post-test (Cronbach’s alpha = 0.79). The Italian translation indicated an acceptable internal consistency reliability for the Indifference subscale both in the pre-test (Cronbach’s alpha = 0.77) and in the post-test (Cronbach’s alpha = 0.73).

#### 2.6.2. Qualitative Measurement

a Focus Group Structure

The main objectives of the focus group were: to deeply explore the students’ perception regarding the impact of arts therapies and psychodramatic methods in the process of death education, and to examine the students’ experience of verbal and artistic elaboration of death fear and its impact upon them. The topics of the focus group were fivefold: (1) the experience of the course (in general); (2) the impact of arts therapies and psychodrama techniques; (3) the experience of verbal and artistic elaboration of the fear of death; (4) the personal meaning of life and death; and (5) the personal representation of death and the emotions associated to it.

b Data Analysis

Quantitative Data Analysis

First, we examined the differences between groups on demographic variables (age, gender, religion, religious level and year of master’s degree), previous experiences (as a formal caregiver to end-of-life clients and whether they had lost someone close to them in the last two years) and baseline scores of study variables. Second, we performed a 2 (time: Time1 and Time2) ×2 (group: Experimental and Control) ANOVA to evaluate the change over time for each examined construct in the two groups and we analyzed the time effect inside each group by test t, adopting the Bonferroni correction to keep the overall type I error under control across the two comparisons. Moreover, we use Cohen’s d as a measure of effect size (d = 0.20 small effect, d = 0.50 medium effect and d = 0.80 strong effect) of the time effect in each group. Power analysis shows an adequate sample size to detect a medium and a small-to-medium effect size of time in each group, 0.98 and 0.79, respectively, in the experimental group and 0.96 and 0.73, respectively, in the control group. The sample size was instead problematic to detect a small effect size of time in both groups, in fact power was, respectively, 0.35 in the experimental group and 0.31 in the control group.

Qualitative Data Analysis

The textual data were analyzed with a qualitative reflexive thematic analysis. Qualitative reflexive thematic analysis is a theoretically flexible method for the recognition of key patterns of meanings and concepts across the dataset [45]. In this method, no preconceptions were implemented, and it is well-suited for studying participants’ experiences and perceptions [46]. The thematic analysis was conducted according to the six phases outlined by Braun and Clarke [45]: familiarization with the data, coding, generating initial themes, reviewing themes, defining and naming themes and writing up the report. Atlas.ti computer software was employed for the qualitative data analysis [47]. All the text files were uploaded on Atlas.ti software and were then analyzed according to the six phases above.

## 3. Results

### 3.1. Quantitative Results

The two groups were similar for all demographic variables, except religious level (t = −7.21 df = 118 *p* < 0.001). Students in the experimental group had lower scores than students in the control group (Table 1). 

Moreover, only three baseline scores of study variables showed differences between the two groups: CCS Total score (t = 5.49 df = 118 *p* < 0.001), CSE Total score (t = 2.58 df = 118 *p* = 0.011) and FATCOD Total score (t = 3.03 df = 118 *p* = 0.003). Students in the experimental group had higher scores than students in the control group. 

ANOVA results showed a significant time x group interaction for the following constructs: TDRS total score F(1,118) = 29.67 *p* < 0.001, DAPR Fear of Death F(1,118) = 17.97 *p* < 0.001, DAPR Death Avoidance F(1,118) = 14.84 *p* < 0.001, DAPR Approach Acceptance F(1,118) = 8.81 *p* = 0.004, CSE total score F(1,118) = 22.15 *p* < 0.001, CS Lesser Indifference F(1,118) = 4.75 *p* = 0.031 and FATCOD Total score F(1,118) = 30.17 *p* < 0.001. Moreover, there was only a significant main effect of time for CCS Total score F(1,118) = 18.33 *p* < 0.001. No differences over time were found for DAPR Neutral Acceptance, DAPR Escape Acceptance and CS Greater Kindness. 

In order to understand the time x group interaction, we examined the change over time in each group (see Table 2). Only students in the experimental group showed a moderate to strong time effect on the TDRS Total score, DAPR Fear of Death, DAPR Death Avoidance, DAPR Approach Acceptance, CSE Total score and FATCOD Total score. In particular, they decreased death representation as annihilation, fear of death and death avoidance and they increased DAPR Approach Acceptance, creative self-efficacy and attitude toward the care of the dying. Both groups showed a small to moderate time effect on the CCS Total score; in fact all students increased their score from Time1 to Time2. Only students in the control group showed a small effect on CS Lesser Indifference; they decreased their score from Time1 to Time2.

### 3.2. Qualitative Results

The thematic analysis showed three themes: (1) the positive impact of the course on death and end-of-life care; (2) the role of the arts therapies on death and end-of-life care themes; and (3) the unhelpful facets of the course.

#### 3.2.1. First Theme: The Positive Impact of the Course on Death and End-of-Life Care Themes

This theme focuses on the positive outcomes that the online course has brought to students regarding the issues of death and end-of-life care.

The first positive impact was a decrease in feelings of unease towards the topics of the course and less feelings of death anxiety as reported by a 40-year-old female student worker:

*“This course has lessened my death anxiety. Is not that I don’t have it anymore now, but now I know that if I die of a progressive disease there is someone who won’t leave me alone, and this helps me when thinking about death. I don’t have negative thoughts anymore, but I’m thinking about having more fun.”*

Students better realized the awareness of the necessity to break down the conspiracy of silence, promoting the expression of truth towards the terminally ill patient. In reference to this, a 26-year-old female student states:

*“This course has allowed me to understand that not being able to speak directly to the patient and make him say “I’m dying, I’m leaving”, I think it leaves unresolved feelings both in the caregivers but also in the patient who dies, it’s bad that the person dies not even being able to talk about what’s happening to him/her with their loved ones.”*

The course raised awareness of the lack of a shared social language and the importance of promoting a discussion on issues related to death, as a 28-year-old female student worker said:

*“Personally, since I started taking this course, I also started talking about it at home [of death], because it’s something that really moved me internally, and I see that it seems that if you start talking a little bit more than before, with a little more serenity, is becoming more acceptable to the other people.”*

Some students have expressed an inefficiency of the health system in taking care of the psychological dimension of those who are dying, as a 23-year-old female student states:

*“I studied in this course all the psychological interventions, everything that can be done for a person who is dying, and I became aware that almost none of this is applied in the health context.”*

The course also allowed students to better understand all the different types of grief, as the 23-year-old female student continues:

*“This course was very helpful for me because it helped me understand a pain that I’m experiencing now related to anticipatory grief, and so I felt not wrong about my feelings, and being able to give it a name is reassuring.”*

The course allowed students to acquire skills that made them feel more capable of dealing with situations involving death and dying and more respectful of the different existential visions of others, as a 23-year-old female student states:

*“Surely if someone were to talk to me about these things, I would feel more capable of dealing with these topics and I also feel more respectful towards different ideas than mine regarding death. I used to be a little ruder about that, but now I feel more respectful of the different ideas that you can have about it.”*

Finally, the course impacted students in their future career choices, stimulating interest in working in the end-of-life field, as the 26-year-old female student worker reports:

*“This course allowed me to understand that I see myself working in the area of end-of-life care in the future.”*

#### 3.2.2. Second Theme: The Role of the Arts Therapies on Death and End-of-Life Care 

This theme takes into consideration the positive peculiarities that the arts therapies have in facing and solving the psychological problems related to death and dying.

The use of arts therapies proved to be a challenge for students, especially those who did not see themselves as creative people. However, they too were able to reveal their creative side, overcoming their personal limits, as a 25-year-old female student says:

*“I have always thought that I was not able to carry out activities like arts therapies, because I am not a creative or extroverted person, I have this limit and I thought I could not express myself in a creative way or in a different dimension from the more pragmatic one. Actually, thanks to the activities, I saw that I didn’t really have these limits and I found a new way of expression.”*

Experiencing the arts therapies firsthand has allowed students to understand the power of finding different methods of expression regarding the topics of death and dying. This made the students feel a sense of liberation from the psychological burdens that the topics of death and dying often carry with them, as the 25-year-old continues, regarding to the photovoice activity:

*“The photovoice, it was the opportunity to put myself more in the game, it was very liberating, because thanks to this activity I realized how much I have inside myself and how much I can’t explain it to myself. The activities opened a bit of a hole inside me that has allowed me to know myself better.”*

Something similar is affirmed by a 28-year-old female worker with regard to the psychodrama workshop:

*“On one hand, putting myself in the shoes of the boy or the guys who were doing it made me relive some personal experiences. But at the end of the activity, I felt a sense of liberation. [...] It was also a physical sensation, during the session it was like a weight was lifted and went away, it was a true relief.”*

The arts therapies workshops have also had a positive impact regarding the processing of emotions related to the topic of death and mourning and the understanding of these emotions, as the 23-year-old female student says:

*“The arts therapies helped me to process the emotions regarding the death topics, they were comforting at times. Listening to the lectures, seeing the material made me say “okay, this thing has a name”, and it made me understand how the emotional processes related to death work.”*

The arts therapies workshops also allowed the students to acquire a greater awareness on the issues of death and dying, as a 24-year-old- female student worker reports:

*“This course has enlightened me. There were a whole series of things of the course that gave me answers to questions that I had about death, to understand and comprehend why I implement some behaviors” and to implement feeling more capable of talking about death, the 25-year-old female student says: “The activities allowed me to realize and speak better about the topics of end-of-life care, even at home with my parents.”*

The students perceived the arts therapy workshops as a safe and secure place to express themselves freely even in relation to a difficult topic such as that of death, as a 56-year-old female student worker states:

*“Having a protected environment, helps you pull out something that is a burden and gives you the opportunity to get rid of some of that weight you have inside”.*

Finally, a further aspect that favored the expression of one’s emotions during and workshops was that of perceiving the closeness of the other companions, as the 56-year-old female continues:

*“I also felt the closeness of the other participants, and therefore I also felt free to tell certain things”.*

#### 3.2.3. Third Theme: The Shortcomings of the Course

This theme highlights the two weaknesses of the course that have been reported by the students.

The first concerns the feelings of anguish and uneasiness during the study of the course topics that was reported by a student who has experienced negative feelings in studying the topics of death and dying; a 28-year-old female says:

*“My contribution is a little discordant from that of others, I must be honest. There were days when I knew I had to go home after work, and doing certain topics was very distressing... Knowing that I had to go home, and still deal with some challenging topics, made me feel a little down, I’m honest. I do not hide that in some moments I felt a little distressed thinking that I had to deal with certain issues.”*

The second shortcoming relates to the arts therapies workshops that caused performance anxiety in students who had never had such an experience before. This performance anxiety concerned both a personal dimension at the beginning of the activity, relating to what extent the student would then be called to open up during the workshop, as a 40-year-old female student worker declares:

*“When I did the practical activities, I was always scared because I thought “Ok, I will do or say what I feel”, but I didn’t know how much I should have shared. This gave me a bit of performance anxiety, I had a lot of emotional fatigue, in managing this aspect.”*

Also, performance anxiety related to the fact of physically entering, with psychodrama activities, in the representation of the contents of the inner world of another person and not feeling up to the situation, as the only male, a 23-year-old student worker, says: “Seeing the emotion in the other made me feel part of their inner world, I don’t know how to say it better. When I was there, there was also some anxiety, performance anxiety that I felt during the activity. I had this performance anxiety because I was afraid of saying or doing something wrong that could harm the person who called me to facilitate the expression of his internal emotions.”

## 4. Discussion

The present study evaluated the impact of an online course regarding death education and palliative psychology on Psychology master’s students in a university located in northern Italy. 

Quantitative results showed that students who participated in the course subsequently reported lower levels of fear of death and death avoidance, feelings that typically can occur when thinking or talking about death and dying [38]. 

The qualitative data were collected in an online setting because of the COVID-19 pandemic, but this factor did not impact the credibility and effect of the death education intervention, as the literature confirms [48,49]. The online environment created a heightened sense of confidentiality in the discussion regarding sensitive issues and allowed all the participants to state their opinions [48]. The online setting was also an effective tool for conducting the focus group as long as the moderators solicited continuous feedback from the group, tested in advance all aspects of the equipment used and supplemented quantitative data with descriptive information as part of a mixed-methods study [49].

Analysis of the qualitative data found that students expressed that they felt less fear and death anxiety. These findings are in line with multiple studies that confirmed the fact that addressing death in death education interventions reduces attitudes of fear or death avoidance [6,7,9,10,18]. 

Other qualitative findings showed decreasing levels of the representation of death as a total annihilation of the self and the personal identity, while there was an increase in the belief in a happy existence after death. From the qualitative findings, similar results were found in participants who expressed that they changed their attitudes about death, perceiving it as more natural, and possessed a better understanding of the feelings related to death and mourning because of the death education course. The literature confirms that death education courses help participants to develop positive attitudes toward death and dying, to develop a view of death as a natural transition at the end of life, and to express themselves with less difficulty on issues related to death and dying [9,12,50].

Quantitative results included an increased inclination to assist people with terminal illnesses, which is also qualitatively confirmed by the increased interest expressed by students in pursuing careers in death education and palliative psychology. These findings are in continuity with a preliminary study investigating psychology students’ interest and sense of confidence in the topics of death education and palliative care [22], also within the European Erasmus+ project Death Education for Palliative Psychology. In the study by Orkibi and colleagues [22], it was found that an interest to work in death education and palliative care fields is influenced by certain factors, such as the lack of knowledge about the issues of death and palliative care or the fear of reliving painful feelings related to personal bereavement in coming in contact with the dying. A death education course, such as the one in the present study, on the other hand, helps to overcome these obstacles by providing the missing theoretical and practical knowledge to adequately handle situations related to death and loss, going to meet the needs noted in the preliminary study [22].

The quantitative results also showed that students participating in the course increased their ability to adapt to new and difficult contexts by finding effective solutions with creative and innovative ideas. This increase in creative self-efficacy can be assumed to be due to the arts therapy workshops, as multiple studies report that arts therapies can stimulate creative responses to difficult situations [51,52,53,54,55]. Qualitative results also show the impact that the arts therapy workshops had on students. They expressed that these workshops greatly facilitated them in expressing themselves regarding the themes of death and dying, in overcoming their expressive limitations and in allowing them to experience a strong sense of liberation. In addition, the workshops enabled them to understand more effectively the emotions related to death and grieving. These results are in accordance with previous studies that report better emotional processing and communication, and a strengthening of one’s expressive and creative skills after the death education course that involved the use of art therapies [12,13].

Other positive elements of arts therapies emphasized by students included the feeling of closeness to other peers and the perception of the workshops as a safe and secure place. These findings are in line with previous research, which asserts that one of the main goals of group art therapy activities is the creation of a welcoming and nonjudgmental environment in which participants can feel free and safe to expose themselves emotionally [56,57,58]. The need to implement university training with death education courses also emerged from the qualitative results. The literature also confirms this need to introduce death education courses in the academic paths of multiple health professions, which are still absent in today’s educational programs in Italy [4,9,59,60]. They could help students acquire the necessary skills to deal competently with death and end-of-life issues and to better manage the fear of death in situations in which they experience it, work-related and otherwise. In this regard, a death education course, such as the one observed in the present study, could help in achieving this goal. In fact, our results show that the death education course consciously and appropriately addressed the issues of death and end-of-life and helped students to approach these issues, to manage the resulting anxiety, and to feel competent in providing their help as future professionals to people who are dying or experiencing bereavement.

### Limitations and Future Directions

The first limitation of the present study concerns the participants. They were part of a convenience sample, thus not randomly selected, and participation was voluntary, since they were all Psychology master’s degree students from the same university in northern Italy. Because of these aspects concerning the participation to the study, the sample presented a gender imbalance, with most of the participants being female. Future studies could expand the sample by assessing the impact of the death education course on other randomly selected students equally distributed between females and males and/or students from other health professions who might come into working contact with the end-of-life field. A second limitation is the size of the sample; future research could expand the sample size of the students involved. As for the qualitative data collection stage, future research could increase the number and duration of focus groups or could conduct detailed individual interviews, in order to delve even deeper into the impact the course had on participants. In addition, future studies could focus more on the impact of the art therapies in the course participants, assessing both the overall impact of the various arts therapies and the impact that each of the arts therapies has in addressing dysfunctional attitudes toward death and the processing of loss.

In conclusion, this study also highlighted the need to implement curricular educational interventions regarding the issues of death and palliative care in professional settings that provide clinical-therapeutic services.

## Figures and Tables

**Table 1:** Descriptive Statistics for Demographic Variables by Group ^1^.

		Experimental Group (N = 64)	Control Group (N = 56)	Group Difference *p*-Value
Age:		22–56; 24.47 (5.36)	22–49; 24.52 (5.41)	0.960
Gender:				0.360
	Male	8 (12%)	4 (7%)	
	Female	56 (88%)	51 (91%)	
	Other	0 (0%)	1 (2%)	
Religion:				0.591
	Christian	30 (47%)	29 (52%)	
	None	34 (53%)	27 (48%)	
	Religious level	1–4; 2.16 (0.86)	1–4; 3.20 (0.70)	<0.001
Formal caregiver to end-of-life clients:				0.057
	No	60 (94%)	56 (100%)	
	Yes	4 (6%)	0 (0%)	
Lost someone close to you in the last two years:				0.180
	No	37 (58%)	39 (70%)	
	Yes	27 (42%)	17 (30%)	
Year of master’s degree:				0.614
	1st	49 (77%)	39 (70%)	
	2nd	14 (22%)	15 (27%)	
	out of course student	1 (2%)	2 (4%)	

^1^ The values reported in the table are the Range, Mean, (Standard Deviation) for continuous variables and frequency, (percentage) for nominal variables. The last column shows the group difference *p*-value computed using the t test for continuous variables and the Chi-square test for nominal variables.

**Table 2:** Change over time examination in the experimental and control groups.

Variable	Experimental Group (N = 64)						Control Group (N = 56)					
	Time 1		Time 2		Time Effect		Time 1		Time 2		Time Effect	
	M	SD	M	SD	t	Cohen’s d	M	SD	M	SD	t	Cohen’s d
TDRS total	21.16	5.03	17.38	5.23	6.45 ***	0.81	20.27	5.34	20.36	5.28	−0.25	−0.03
DAPR Fear of Death	3.31	0.94	2.84	0.85	5.38 ***	0.67	3.32	0.91	3.30	0.92	0.33	0.04
DAPR Death Avoidance	2.46	0.99	1.98	0.77	5.79 ***	0.72	2.79	0.99	2.76	1.00	0.29	0.04
DAPR Neutral Acceptance	3.93	0.58	4.06	0.55	−2.24	−0.28	3.82	0.55	3.81	0.64	0.25	0.03
DAPR Approach Acceptance	2.05	0.74	2.29	0.78	−3.20 **	−0.40	2.24	0.84	2.22	0.85	0.52	0.07
DAPR Escape Acceptance	2.43	0.70	2.30	0.91	1.31	0.16	2.55	0.84	2.54	0.93	0.17	0.02
CCS Total	13.94	3.66	15.20	4.22	−4.01 ***	−0.50	10.61	2.87	11.16	3.07	−2.01	−0.27
CSE Total	13.02	5.11	16.63	3.55	−6.53 ***	−0.82	10.77	4.33	10.86	4.05	−0.18	−0.02
CS Greater Kindness	17.38	2.07	17.63	1.87	−1.00	−0.12	16.96	2.10	16.89	1.88	0.35	0.05
CS Lesser Indifference	6.86	2.09	6.64	2.06	0.89	0.11	6.96	2.07	7.46	2.21	−2.33 *	−0.31
FATCOD Total	3.89	0.32	4.12	0.30	−7.56 ***	−0.95	3.71	0.32	3.72	0.31	−0.35	−0.05

* *p* < 0.05; ** *p* < 0.01; *** *p* < 0.001.

## Data Availability

The data that support the findings of this study are available from the corresponding author, L.R., upon reasonable request.
